# The role of ZFP57 and additional KRAB-zinc finger proteins in the maintenance of human imprinted methylation and multi-locus imprinting disturbances

**DOI:** 10.1093/nar/gkaa837

**Published:** 2020-10-14

**Authors:** Ana Monteagudo-Sánchez, Jose Ramon Hernandez Mora, Carlos Simon, Adam Burton, Jair Tenorio, Pablo Lapunzina, Stephen Clark, Manel Esteller, Gavin Kelsey, Juan Pedro López-Siguero, Guiomar Perez de Nanclares, Maria-Elena Torres-Padilla, David Monk

**Affiliations:** Imprinting and Cancer group, Bellvitge Institute for Biomedical Research, Gran via, L’Hospitalet de Llobregat, Barcelona, Spain; Imprinting and Cancer group, Bellvitge Institute for Biomedical Research, Gran via, L’Hospitalet de Llobregat, Barcelona, Spain; Department of Obstetrics and Gynecology, Valencia University and INCLIVA, Valencia, Spain; Department of Obstetrics and Gynecology, BIDMC, Harvard University, Boston, MA, USA; Institute of Epigenetics and Stem Cells, Helmholtz Zentrum München, München, Germany; Medical and Molecular Genetics Institute, University Hospital La Paz, Madrid, Spain; CIBERER, Centro de Investigacion Biomedica en Red de Enfermedades Raras, ISCIII, Madrid, Spain; Medical and Molecular Genetics Institute, University Hospital La Paz, Madrid, Spain; CIBERER, Centro de Investigacion Biomedica en Red de Enfermedades Raras, ISCIII, Madrid, Spain; ITHACA, European Reference Network on Rare Congenital Malformations and Rare Intellectual Disability; Epigenetics Programme, The Babraham Institute, Babraham, Cambridge, UK; Josep Carreras Leukeamia Research Institute, Can Ruti, Cami de les Escoles, Badalona, Barcelona, Spain; Department of Physiological Sciences II, School of Medicine, University of Barcelona, Barcelona, Catalonia, Spain; Institucio Catalana de Recerca i Estudis Avançats (ICREA), Barcelona, Catalonia, Spain; Centro de Investigacion Biomedica en Red Cancer (CIBERONC), Madrid, Spain; Epigenetics Programme, The Babraham Institute, Babraham, Cambridge, UK; Centre for Trophoblast Research, University of Cambridge, UK; Servicio de Endocrinología Pediátrica, Hospital Carlos de Haya, Málaga, Spain; (Epi)Genetics Laboratory, BioAraba Research Health Institute, Araba University Hospital, Vitoria-Gasteiz, Alava, Spain; Institute of Epigenetics and Stem Cells, Helmholtz Zentrum München, München, Germany; Imprinting and Cancer group, Bellvitge Institute for Biomedical Research, Gran via, L’Hospitalet de Llobregat, Barcelona, Spain; Biomedical Research Centre, University of East Anglia, Norwich Research Park, Norwich, UK

## Abstract

Genomic imprinting is an epigenetic process regulated by germline-derived DNA methylation that is resistant to embryonic reprogramming, resulting in parental origin-specific monoallelic gene expression. A subset of individuals affected by imprinting disorders (IDs) displays multi-locus imprinting disturbances (MLID), which may result from aberrant establishment of imprinted differentially methylated regions (DMRs) in gametes or their maintenance in early embryogenesis. Here we investigated the extent of MLID in a family harbouring a *ZFP57* truncating variant and characterize the interactions between human ZFP57 and the KAP1 co-repressor complex. By ectopically targeting ZFP57 to reprogrammed loci in mouse embryos using a dCas9 approach, we confirm that ZFP57 recruitment is sufficient to protect oocyte-derived methylation from reprogramming. Expression profiling in human pre-implantation embryos and oocytes reveals that unlike in mice, *ZFP57* is only expressed following embryonic-genome activation, implying that other KRAB-zinc finger proteins (KZNFs) recruit KAP1 prior to blastocyst formation. Furthermore, we uncover ZNF202 and ZNF445 as additional KZNFs likely to recruit KAP1 to imprinted loci during reprogramming in the absence of ZFP57. Together, these data confirm the perplexing link between KZFPs and imprint maintenance and highlight the differences between mouse and humans in this respect.

## INTRODUCTION

Genomic imprinting in mammals refers to a highly-coordinated epigenetic mechanism that leads to parental allele-specific gene expression (1). Imprinted genes are known to play critical roles in fetal development, placenta and brain function, which when perturbed, result in imprinting disorders (IDs) (2). The characteristic monoallelic expression of imprinted genes is largely conferred by allele-specific DNA methylation, referred to as differentially methylated regions (DMRs) that act as imprinting control regions (ICRs). The differential DNA methylation at the majority of ICRs is inherited from the gametes and maintained in pre-implantation embryos during global epigenetic reprogramming (3). The resulting germline-derived DMRs (gDMRs) in general are ubiquitously present in all tissues later in development, but a subset have a tissue-specific profile, often restricted to the placenta (4,5). In addition to gDMRs, a variety of mechanisms ensure monoallelic expression of imprinted transcripts, including somatically acquired DMRs (also known as secondary DMRs) and allele-specific histone modifications, including H3K9me3 and H3K27me3 (6,7). Intriguingly, a number of tissue-specific imprinted genes, whose temporal imprinting is limited to cleavage stage embryos and placenta, appear to utilize non-canonical mechanisms not associated with gDMRs but reliant on histone modifications (8–10).

Gene encoding Kruppel-associated (KRAB)-zinc finger proteins (KZFPs), which are involved in the establishment and maintenance of repressive chromatin states, are highly abundant in the mammalian genome (11). KZFPs have classically been linked to the silencing of transposable elements, with underlying sequence-specificity attributed to unique combinations of zinc-finger that target different retrotransposon families (12). Once recruited to a sequence, transcriptional repression is mediated by the recruitment of the KAP1 co-repressor complex, which induces the local deposition and maintenance of H3K9me3 (13). KZFPs have been shown not only to recognise transposable elements, but an increasing number of these proteins also bind to single-copy regions in the genome. One of the most-studied members in this respect is ZFP57, which in addition to Intracisternal A Particle (IAP) Long Terminal Repeats (LTR) retrotransposons, also binds to imprinted gDMRs (14–17). Because ZFP57 is highly abundant in mouse oocytes and recognises methylated CpG dinucleotides in the TGCC^me^GC hexamer consensus motif, this KZFP is largely responsible for maintaining methylation at the majority of gDMRs during mouse pre-implantation reprogramming (15,18,19). The high oocyte expression of *Zfp57* is consistent with it being a maternal-zygotic effect gene in mice. When expression is depleted in zygotes derived from heterozygous females (*Zfp57* –/– offspring from *Zfp57*+/– mothers) the severity of both methylation defect and penetrance of the phenotype are less severe than in homozygous nulls from *Zfp57* –/– mothers (16). Recessive mutations in the human orthologue causes transient neonatal diabetes mellitus (TNDM) associated with multi-locus methylation disturbance (MLID) (20), a phenomenon in which loss-of-methylation (LOM) at numerous imprinted DMRs is observed in addition to the disease-associated loci (2). To date, 14 families have been described with either missense or truncating mutations in *ZFP57* (21,22), all have a notably similar methylation signature; complete hypomethylation of the *PLAGL1* DMR (*PLAGL1*:alt-TSS-DMR) and variable combinations of mosaic hypomethylation at *GRB10* (*GRB10*:alt-TSS-DMR), *PEG3* (*PEG3*:TSS-DMR), *MEST* (*MEST*:alt-TSS-DMR), *NAP1L5* (*NAP1L5*:TSS-DMR) and *GNAS* (*GNAS-AS1*:TSS-DMR). However, whether ZFP57 can directly regulate those loci has not been addressed.

In this study, we investigate the stability of mosaic hypomethylation at imprinted DMRs in a family with a *ZFP57* truncating mutation and characterize ZFP57-KAP1 functional interactions in humans. Furthermore, we identify additional KZFPs that are expressed at the appropriate time during human development that have the ability to recruit the KAP1. Together, these results shed light on the species-specific function of ZFP57 in regulating imprinting maintenance during development.

## MATERIALS AND METHODS

### Ethics

The family with the ZFP57^p.E^^123*^ mutation provided informed consent to be involved in this study. The ethical approval for this aspect of the work was granted by the Bellvitge Institute of Biomedical Research Ethics Committee (PR096/10), the Basque Ethics Committee (IRB#PI2013214 and PI2017018) and Hospital Universitario La Paz (IRB-HULP PI446). Collecting placenta samples was approved by the Bellvitge Institute of Biomedical Research Ethics Committee (PR006/08). The use of surplus human oocytes and embryos for this study was evaluated and approved by the scientific and ethic committee of the Instituto Valenciano de Infertilidad (IVI) (1310-FIVI-131-CS), Centro de Medicina Regenerativa de Barcelona (CMRB), the National Committee for Human Reproduction (CNRHA) and the Regional Health Departments for Valencia and Catalyuna (10/2017). Ethical approval for the using hES cells was granted by the Bellvitge Institute for Biomedical Research Ethics Committee (PR096/10) and Comites Éticos de Investigación Clínica (CEIC) del Centro de Medicina Regenerativa de Barcelona-CMR[B] (28/2012) and complied with the legal guidelines outlined by the Generalitat de Catalunya and the Conselleria de Salut. Mouse work was approved and performed under the authorization of the Upper Bavarian authorities. Animal husbandry and breeding were conducted according to the institutional guidelines for the care and the use of laboratory animals.

### Patient samples

A family with siblings diagnosed with TNDM who were referred to the paediatric endocrinology at Carlos de Haya Hospital (Malaga, Spain) were used in this study. The previously identified mutation was confirmed in proband II.2 using standard PCR and sequencing as described by Court and colleagues (23). Two separate cohorts of MLID patients were screened for mutations, the first comprising of mainly Beckwith–Wiedemann syndrome patients were from the Spanish overgrowth registry (*n* = 28), while patients with Pseudohypoparathyroidism type 1B (PHP1B; *n* = 10) and TNDM (*n* = 3) were from the BioAraba Research Health Institute. A collection of 15 human leukocyte and buccal swabs were collected from volunteers at PEBC-IDIBELL and used as controls to determine the normal range of methylation at imprinted DMRs. Twelve human metaphase II oocytes and a single surplus human blastocyst were obtained from the Instituto Valenciano de Infertilidad (FIVI) (Valencia, Spain). Vitrified oocytes were thawed using a kit for de-vitrification (Kitazato Valencia, Spain) following manufacturer's instructions.

### Single cell lymphoblastoid colony expansion

Isolated clonal cell lines were generated by dilution plating of a polyclonal lymphoblastoid cell as previously described (24). Transformation with Epstein–Barr virus (EBV) was performed on a fresh blood sample from patient II.1. Isolated B-cells within the sample were immortalized with the supernatant of the EVB producer cell line B95.8. Surviving colonies were propagated to a density sufficient for DNA isolation.

### Methylation array hybridisation

We generated methylation datasets for affected family members with the ZFP57^p^^.E^^123*^mutation using the Illumina Infinium HumanMethylation450 (HM450k) and Infinium HumanMethylationEPIC (EPIC) BeadChip arrays. Bisulphite conversion of 600 ng of DNA was performed according to the manufacturer's recommendations for the Illumina Infinium Assay (EZ DNA methylation kit, ZYMO, Orange, CA, USA). The bisulphite-converted DNA was used for hybridisation following the Illumina Infinium HD methylation protocol at genomic facilities at IDIBELL (Barcelona, Spain). In addition to these samples, we also utilized our control leukocyte datasets from GSE103738 and GSE149572 (25).

### Statistical analyses of the Illumina arrays

Raw data was normalised using the default control probes in BeadStudio (version 2011.1) Infinium HD), subtracting signal background and correcting for interpolate variation. Probes with a detection *P*-value >0.01, containing SNPs within the integration or extension bases as well as those with potential cross-reaction due to multiple sequences homologies were discarded. Probes lacking signal values in one or more of the analysed samples were discarded also. For the analysis of known imprinted domains, probes mapping to the DMRs identified by Court *et al.* and Hernandez Mora *et al.* were directly examined (25,26).

### Allele-Specific bisulphite PCR

For standard bisulphite conversion, we used the EZ DNA Methylation-Gold kit (ZYMO, CA, USA) according to the manufacturer's instructions. Approximately 2 μl of bisulphite-converted DNA was used in each amplification reaction catalysed by Immolase Taq polymerase (Bioline). The resulting PCR product was sub-cloned into the pGEM-T easy vector (Promega) for sequencing (for primer sequences see Supplementary Table S1). The EZ DNA Methylation-Direct kit (ZYMO), in which cell lysates are subject to direct bisulphite conversion, was used for the methylation profiling of individual blastocysts and pooled oocytes. Nested bisulphite PCR was required to obtain amplicons for sub-cloning due to the small amounts of available samples.

### Pyrosequencing analysis for methylation quantification

Approximately 50 ng of bisulphite converted DNA was used for PCR prior to pyrosequencing. Standard bisulphite PCR was used to amplify the imprinted DMRs with the exception that one primer was biotinylated (see Supplementary Table S1 for primer sequences). In all cases the entire biotinylated PCR product (diluted to 40 μl) was mixed with 38 μl of Binding buffer and 2 μl (10 mg/ml) streptavidin-coated polystyrene beads. After washing in 70% ethanol, DNA was denaturated with 50 μl 0.5M NaOH. The single-stranded DNA was hybridized to 40-pmol sequencing primers dissolved in 11 μl annealing buffer at 80°C. For sequencing, forward primers were designed to the complementary strand. The pyrosequencing reaction was carried out on a PyroMark Q96 instrument. The peak heights were determined using Pyro Q-CpG1.0.9 software (Biotage).

### Nested multiplex bisulphite PCR

We employed a multiplex nested PCR approach to maximise data generation from individual mouse embryos which were subject to direct bisulphite conversion. The protocol has been described elsewhere (27); essentially two sets of primers (outer and inner pairs) were designed to each locus and robustly optimized to ensure efficient amplification of both methylated and unmethylated strands at a single annealing temperature without contamination or the formation of primer dimers (primers detailed in Supplementary Table S1). For the multiplex step, outer primers (four separate pairs targeting different loci) were co-amplified in the first reaction using Immolase Taq polymerase (Bioline) for 45 cycles. The multiplex PCR was carried in a total volume of 97 μl, including the 30 μl of embryo converted DNA as a template. We included two controls: blank with no sample and 10 ng of BS-converted DNA as a positive control. The nested second round amplifications utilized the internal primer pairs specific for each region with individual PCRs performed on aliquots of the first-round product. The nested reaction was performed in a total volume of 25 μl reaction using Immolase Taq polymerase (Bioline). These were amplified for 45 cycles and the resulting amplicons used for pyrosequencing or sub-cloned into the pGEM-T easy vector.

### Single colony restriction enzyme-based analysis of methylation

To analyse the methylation state of four different regions in the lymphoblastoid single colonies we performed a protocol similar to the one described by Cheow and colleagues (28). After collecting the DNA of each of the clonal lymphoblastoid population, the DNA was digested with the methylation-sensitive enzyme BstUI for 2 h at 37°C in a thermocycler. The digested DNA was subjected to amplification by PCR using primers flanking a restriction site in the region of interest, and the methylation state was identified depending on the size of the amplicon, larger amplicons were methylated and small amplicon resulted irrespective of methylation status (primer sequences are detailed in Supplementary Table S1).

### Genotyping and imprinting analysis

Potential SNPs in *ZFP57* exons and hap-ASM were identified in the UCSC hg19 browser and genotyped by PCR and direct sequencing in hES cell lines. Sequence traces were interrogated using Sequencher v4.6 (Gene Codes Corporation, MI) to distinguish heterozygous and homozygous samples. Heterozygous sample cell lines were analyzed for allelic expression using RT-PCR and bisulphite PCR, incorporating the polymorphism within the final PCR amplicon (see Supplementary Table S1 for primer sequences).

### Quantitative Real-Time-PCR

Expression of *ZFP57* isoforms, *ZNF202* and *ZNF445* transcripts were analyzed using a fluorochrome (SYBR^®^ Green)-based quantitative real-time RT-PCR assay and normalized against *RPL19*. cDNA was synthesized as previously described (29). All assays were run in triplicate in 96-well or 384-well plates using Real-Time-PCR System (Applied Biosystems). Only samples with two or more valid readings per triplicate were included in our analysis. Dissociation curves were generated at the end of each reaction to rule out the presence of primer-dimers or unexpected DNA species in the reaction. Results were analyzed with the SDS 2.3 software (Applied Biosystems).

### Antibodies

The primary antibodies used included α-HA (Sigma H6900; co-IP 5 μg; WB 1:1000), α-FLAG (Sigma 180, 5 μg; WB 1:1000 ChIP 5 μg), α-myc (Sant Cruz Biotech 9E10, 5 μg; WB 1:5000), α-V5 (Bio-Rad MCA1360, co-IP 5 μg; WB 1:1000) and α-B actin (Sigma, A3854, WB 1:1000). The secondary antibodies used were α-mouse (Sigma A9044) and α-rabbit (Sigma A0545)

### Transfection, protein extraction and immunoprecipitation

All cell lines were grown in DMEM (Life Technologies) supplemented by 10% fetal bovine serum (Life Technologies). Cells were routinely tested for mycoplasm infection with PCR- detection kit (Biotools-Biot. & Med). All transfections for mammalian over-expression were carried out using PEI reagent following supplier's recommendations. Transfections with Cas9 utilized OptiMEM media (Gibco) and Lipofectamine 3000 (ThermoFisher) using recommended protocols.

Nuclear and cytoplasm extract were prepared for protein extraction to perform western blot and co-immunoprecipitation assays. Cell were pelleted and washed twice with cold PBS, and proteins were extracted according to the Dignam protocol: first extraction for the cytoplasmic soluble fraction was made with Buffer A (10 mM Tris pH 7.8; 10 mM KCl; 1.5 mM MgCl_2_) and second extraction for the nuclear soluble fraction was made with Buffer C (10 mM Tris pH 7.8; 0.42 M NaCl; 1.5 mM MgCl_2_; 0.2 mM EDTA; 25% glycerol). Extracts were kept at −80°C until use.

For immunoprecipitation experiments, cell extracts were incubated with either α-HA resin or α-FLAG, α-V5 or α-Myc tag antibody crosslinked to protein G-magnetic beads (ThermoFisher), overnight. Beads were washed three times with BC100 buffer (10 mM Tris pH 7.8, 0.5 mM EDTA, 0.1 mM phenylmethylsulfonyl fluoride, 0.1 mM dithiothreitol (DTT), 10% glycerol, 100 mM KCl) and three times with BC500 buffer (500 mM KCl). Then, proteins were eluted with 0.2 M Glycine pH 2.3.

### Western blotting

Protein extracts and co-IPs were mixed with 5× SDS loading buffer (1 M Tris pH 7.8, 87% glycerol, 10% SDS and 0.1 g of bromophenol blue), and samples were boiled for 5 min at 95°C. Samples were loaded on a gel along with 5 μl of pre-stained PageRuler protein ladder (Fermentas) and run at 25 mA. The gels were blotted with transfer buffer (Tris 0.25M, glycine 1.92 M and 1% SDS) on to nitrocellulose membrane in a blotting chamber (Bio-Rad) for 45 min at 0.40 A. Successful transfer of proteins was confirmed by Ponceau (Sigma) staining. Then, membrane was blocked with 5% low fat milk (Merck) diluted in PBS-Tween for 1 h at room temperature. The membrane was subsequently washed three times in PBS–Tween and incubated with the primary antibody, in 5% low fat milk overnight at 4°C on a horizontal agitator. Afterwards, membrane was washed three times for 5 min with PBS-Tween, then the corresponding peroxidase-conjugated secondary antibody was applied in 5% low fat milk dilution 1:5000 for 1 h at room temperature. The membrane was washed with PBS–Tween three times more and placed on a developing machine.

### Histone peptide array

MODified™ Histone peptide Array (Active Motif) containing 384 different histone modification combinations was used. The array was blocked overnight in 5% low fat milk (Merck) diluted in TBS–Tween solution, followed by three washes for 5 min with TBS–Tween and incubated overnight in an orbital shaker with 6 μM of the corresponding purified protein in TBS–Tween. The array was washed three times in TBS–Tween and incubated with α-HA primary antibody, in 5% low fat milk for 3 h on a horizontal blocker at 4°C. Following three more washes in TBS-Tween, α-rabbit peroxidase-conjugated secondary antibody was applied in 5% low fat milk dilution 1:2500 for 1 h at room temperature. Following three last washes in TBS–Tween the array filters were develop and quantified.

### dCas9-fusion plasmids, *in vitro* transcription, zygote microinjections and embryo culture

The dCas9-Zfp57[KRAB]-T2A-GFP fusion construct was generated using the pLV hUbC-dCas9-VP64-T2A-GFP plasmid backbone (30). The NheI sites were used to remove VP64 and introduce mouse ZFP57 (amino acids 13–55 encoding the KRAB domain). We also generated the control plasmid, dCas9-control, with no insert (Nhe1 self-ligation). *In vitro* transcription (IVT) was utilized to generate full-length RNA for zygote injections using the mMESSAGE mMACHINE^®^ T7 Ultra Kit (Thermofisher). The IVT template was the PCR product generated using the dCas9-Zfp57[KRAB]-T2A-GFP construct amplified with a forward primer encompassing the T7 RNA polymerase promoter upstream of the dCas9 sequence and a reverse primer with a stop codon and polyA tail designed to the end of the GFP (primers detailed in Supplementary Table S1). The *in vitro* transcription, *DNA*seI treatment and LiCl clean-up were performed following the suppliers recommendations. The resulting IVT RNA was resuspended in TE and quantified by spectrophotometer (Nanodrop). To ensure IVT products were full-length without degradation, an aliquot of IVT RNA was visualized following gel electrophoresis on a 1.5% agarose gels prepared under MOPS buffer/formaldehyde denaturing conditions. Embryos were collected from 5 to 7 week-old F_1_ (C57BL/6J × CBA/H) super-ovulated females crossed with F_1_ males. Superovulation was induced by intraperitoneal injection of pregnant mare serum gonadotropin (PMSG, Intervet, 5 IU) and human chorionic gonadotropin (hCG, Intervet, 7.5 IU) 46–48 h later. Early zygotes were collected at 18h hCG injection and microinjected with 1–2pl of 200 ng/μl dCas9-Zfp57[KRAB]-T2A-GFP mRNA, 20 ng/μl of crRNA and 20 ng/μl of tracrRNA. Embryos were cultured in K-modified simplex optimized medium microdrops under oil at 37°C, 5% CO_2_ for 78 h until the blastocyst stage.

### Oocyte and embryo RNA-seq analysis

The single-cell (sc) RNA-seq libraries were generated using the Nextera XT kits on previously amplified cDNA (https://doi.org/10.1038/nprot.2014.006). After sequencing, reads were trimmed using Trim Galore, with a minimal ‘phred’ score for base of 13 and a minimum allowed read length of 35 bases (as recommended by https://doi.org/10.1016/j.cell.2015.10.039). Remaining reads were mapped by Hisat2 against the GRCh38 human genome. Reads were assigned to transcripts present in the 94 Ensembl annotation version of the GRCh38 human genome by using the Rsubread R package (https://doi.org/10.1093/nar/gkt214). Cells with <0.75 million or >10 million assigned reads were filtered out, as well as cells with >7500 detected transcripts. Transcripts per million (‘TPM’) normalisation was also performed. Expression plots for pseudo-merged individual embryos were created using in-house R scripts.

### KZNF ChIP-seq data analysis

Public ChIP data was obtained from GEO data series GSE78099 (12). Files with peaks processed score signals were retrieved and the data plotted using in-house R scripts.

## RESULTS

### Characterization of methylation defects in TNDM-MLID siblings with homozygous ZFP57^p.E^^123*^

**Figure 1.**
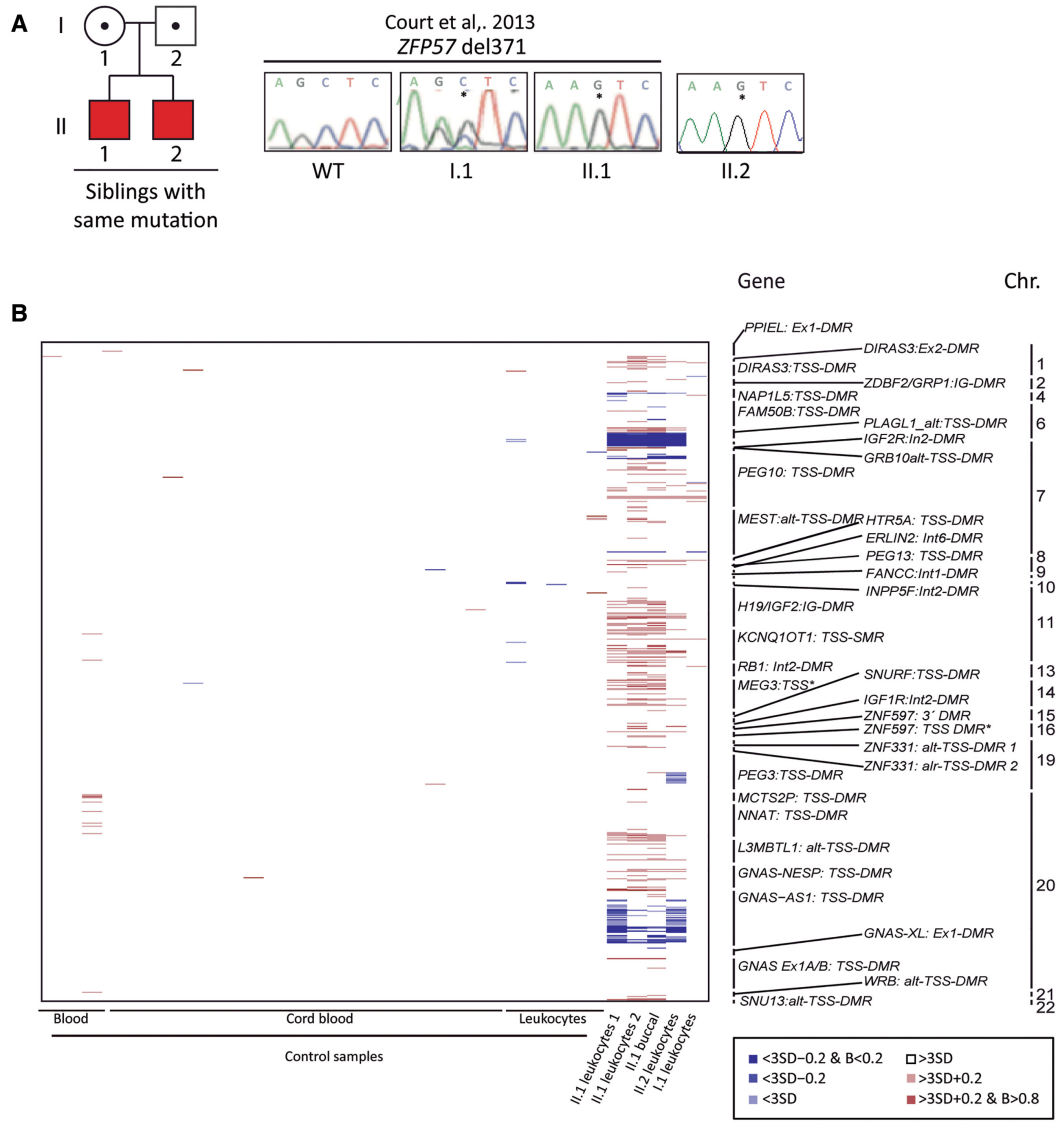
Methylation profiling in a family with TNDM-MLID caused by *ZFP57* mutation. (**A**) Family pedigree with transmission of the ZFP57^p.E^^123*^ and examples of the sequence electropherograms showing the mutation in I.1, II.1 and control samples. (**B**) Analysis of 36 ubiquitously imprinted DMRs using Infinium HumanMethylationEPIC BeadChip arrays. Methylation differences are color coded according to severity, with blue and red representing hypo- and hypermethylation, respectively.

We previously reported a child with TNDM (II.1), who we found was homozygous for a one base-pair deletion (p.E123*) in *ZFP57*, inheriting the mutations from both heterozygous parents. Methylation profiling at diagnosis revealed severe hypomethylation at *PLAGL1* and mosaic LOM at *GRB10, NAP1L5* and *GNAS*-XL DMRs in II.1, but not in the carrier mother (23) (Figure 1). Ten years after the first child, a second sibling (II.2) was born with comparable TNDM and birthweight, but lacked several of the additional clinical features observed in II.1 (Supplementary Table S2). Upon molecular investigation this child was shown to have the same homozygous deletion (Figure 1). Using the EPIC methylation arrays and bisulphite PCR with pyrosequencing, we confirmed a hypomethylated signature similar to the sibling at ubiquitous imprinted DMRs (Spearman's correlation rho = 0.85, *P* = 0), with the exception of additional hypomethylation at the *PEG3* DMR. (Figure 1; Supplementary Figure S1A; Supplementary Table S3).

To further characterise the stability of the imprinting defects in the two children and understand the potential role of ZFP57^p.E^^123*^ in tissue-specific mosaicism, we hybridised buccal-derived DNA and a second leukocyte sample from II.1 to the EPIC arrays. When comparing the two blood-derived samples we observed that the hypomethylation signature is stable over time (Spearman's correlation rho = 0.89, *P* = 0), with the *PLAGL1* DMR being the most severely affected consistent with this being the disease-causing locus. The two sibling samples differed from the unaffected mothers sample in their imprinted methylation profile (Spearman's correlation rho = 0.53, *P* = 0). Furthermore, the hypomethylation also occurred in buccal samples, with the same imprinted DMRs affected to a similar extent (Spearman's correlation rho = 0.86, *P* = 0) (Supplementary Figure S1A).

**Figure 2.**
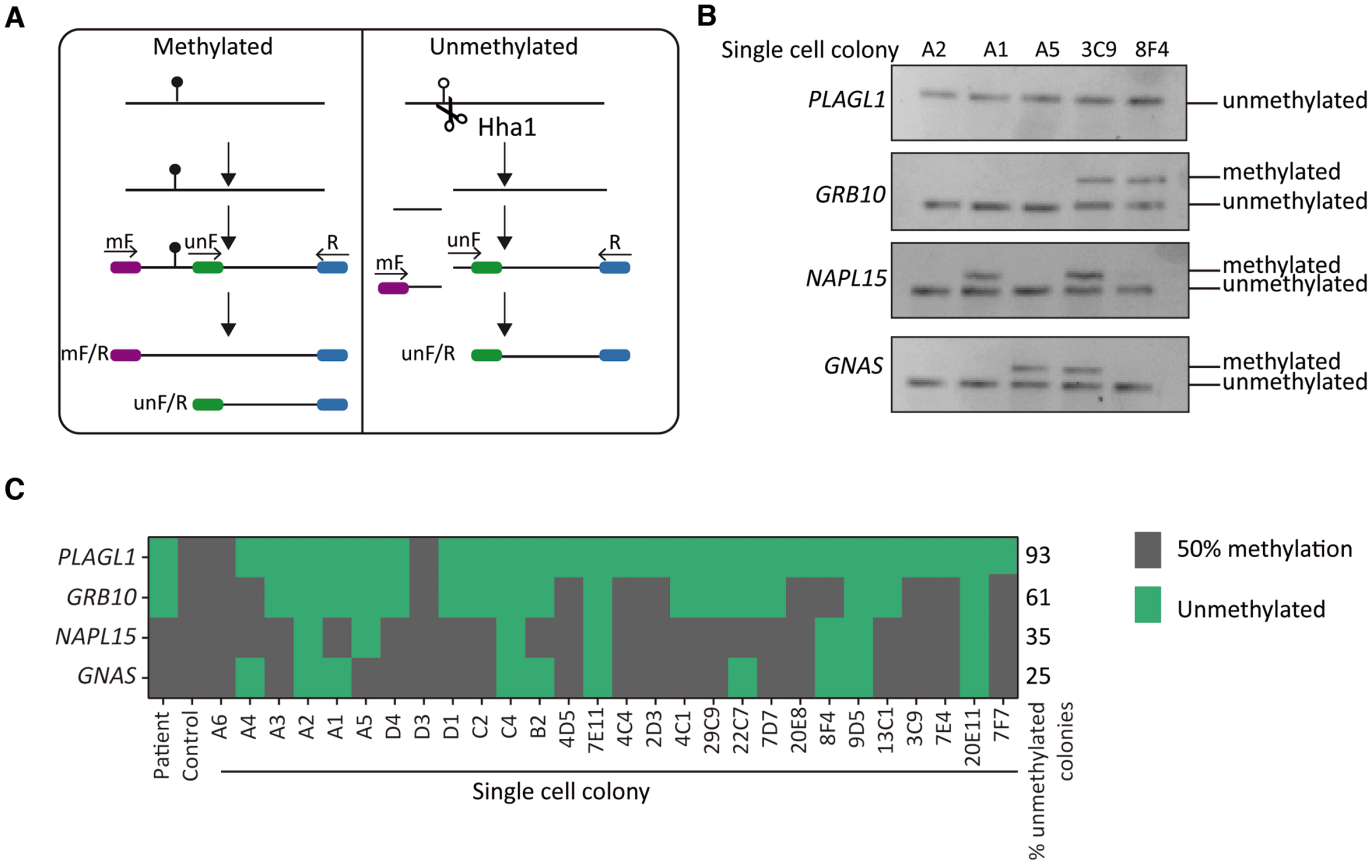
Characterising the level of mosaic methylation through analysis of single cell-derived clones. (**A**) Schematic overview of the modified SCRAM technique. (**B**) Examples of the resulting PCRs for representative colonies. (**C**) Methylation results for all colonies analysed with the colony ID indicated and the investigated imprinted DMR labelled on the left.

Hypomethylation associated with MLID is often partial, with distinct loci subject to varying degrees of LOM. To understand this mosaic pattern and why some regions are only partially affected, we generated a lymphoblastoid cell line following Epstein-Barr virus (EBV) transformation from the second blood sample of II.1. Initial characterisation upon EBV transformation showed a stable methylation pattern indicating that the immortalization process had not induced additional changes at imprinted gDMRs (Supplementary Table S3). We subsequently utilized this line to generate 28 expanded colonies (<500 cells) derived from single cells, which we used to characterize imprinted methylation using a modified SCRAM technique (28) (Figure 2A, B). This revealed *PLAGL1* LOM in 93% (26/28) of lines, whilst *GRB10* hypomethylation was observed in 61% (17/28) of the lines and in 35% (10/28) and 25% (7/28) for *GNAS*-XL and *NAP1L5*, respectively (Figure 2C). These results are consistent with the detected levels of mosaicism observed using the Infinium arrays. Interestingly, while the lines varied with respect to hypomethylation at *GRB10, GNAS* and *NAP1L5* varied, we never observed hypomethylation of these loci independent of *PLAGL1*, and lines with normal *PLAGL1* also had normal methylation at the other DMRs. Together these results indicate that the methylation signature associated with recessive mutations of *ZFP57* in humans differ significantly from the equivalent zygotic mutations in mouse.

### Determining the histone modification binding preference for the ZFP57-KAP1 co-repressor complex

One possible explanation why only selected gDMRs are affected when *ZFP57* is mutated could be due to preferential binding to genomic regions with specific patterns of histone modifications. The TGCC^me^GC consensus motif is present in the human genome >100 000 times, with ∼62% of these mapping to loci with >75% methylation, yet ChIP-seq data reveals ∼3000 binding sites (12), of which only selected gDMRs are affected when the gene is mutated. To determine whether ZFP57 recognises any post-translation histone modification, adding an additional layer of the complexity to the recognition of the binding motifs within gDMRs, we purified HA-ZFP57 and HP1γ-HA, a chromodomain containing member of the KAP1 co-repressor complex, from transiently transfected cells and exposed the proteins separately to MODified™ histone peptide arrays. These experiments conclusively revealed that ZFP57 is unable to directly bind to any histone modification, consistent with the absence of chromo-domains, whilst HP1γ robustly binds to H3K9me2 and H3K9me3 (Supplementary Figure S2). This suggests that in addition to direct sequence recognition, the ZFP57-KAP1 co-repressor complex associates with these repressive histone marks, which decorate most gDMRs, via the recruitment by HP1γ.

### Human ZFP57 interacts with DNMT3 and KAP1 co-repressor complex

**Figure 3.**
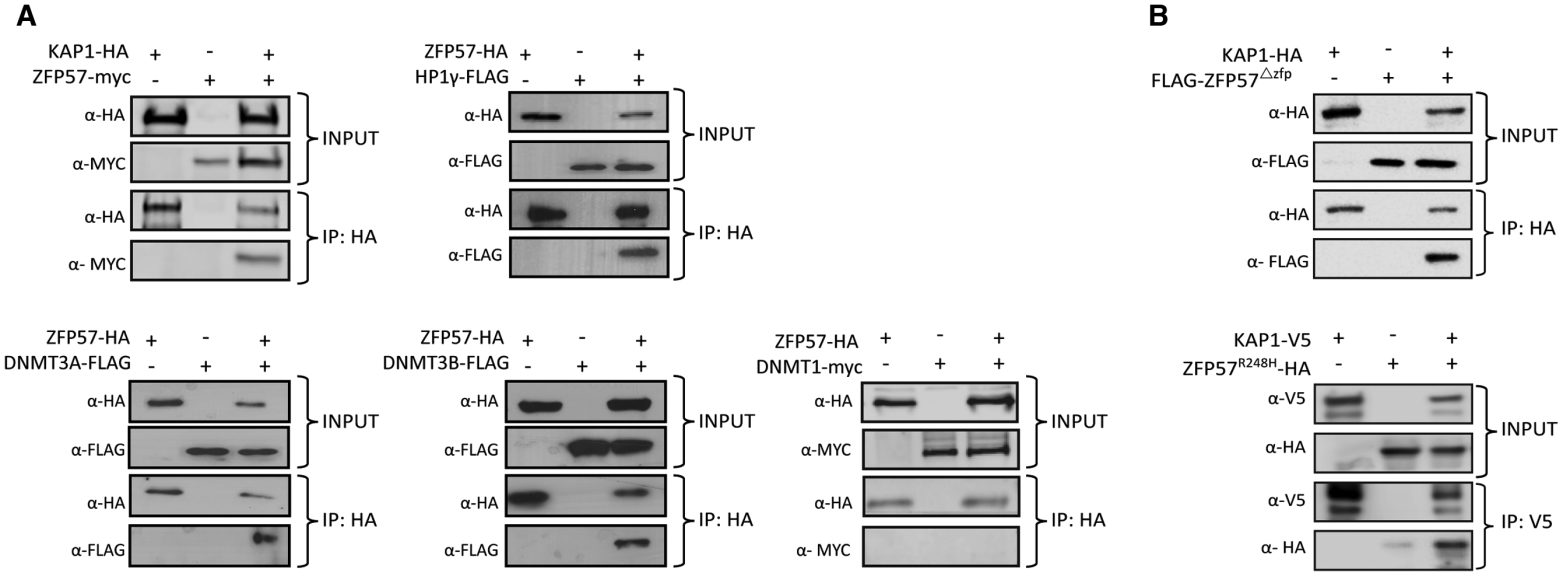
Characterizing *in vivo* protein-protein interactions for ZFP57 and member of the KAP1 co-repressor complex. (**A**) Western blotting of co-immunoprecipitated material from tetracycline induced ZFP57-HA and transiently transfected interacting partners including epitope tagged KAP1, HP1γ, DNMT3A, DNMT3B and DNMT1. (**B**) Co-immunoprecipitation and western blotting for transiently transfected FLAG-ZFP57^Δznf^ with KAP1-HA and HA-ZFP57^p.R228H^ with KAP1-V5.

Previous studies have shown that mouse ZFP57 physically interacts with DNMT1, DNMT3A/B and KAP1 in mouse embryonic stem cells (14,15). Since mouse and human ZFP57 only share 37% amino-acid identify, we wanted to address whether the human orthologue was able to interact with the KAP1 co-repressor complex. To test this physical interaction, we generated full-length ZFP57 epitope tagged inducible over-expressing cell lines based on the T-REx 293 system to overcome the lack of endogenous expression in cell lines. In ZFP57-myc and KAP1-HA expressing cells, antibodies against the HA epitope were used to pull-down KAP1 associated proteins and myc antibody was used to probe the immunoprecipitated material (Figure 3A). ZFP57-myc was detected when it was over-expressed with KAP1-HA confirming that these proteins interact as observed in mice. Similar co-IP experiments revealed that HP1γ-FLAG also interacts physically with ZFP57-HA in our T-REx 293 cells. Furthermore, FLAG-tagged DNMT3A and 3B could also interact with ZFP57-HA, although no co-immunoprecipitation was observed with DNMT1 indicating that ZFP57 maintains allelic methylation by recruiting the *de novo* methyltransferases (Figure 3A).

To determine the effect of the ZFP57^p.E^^123*^on the recruitment of the KAP1 co-repressor complex, we generated a truncated version of *ZFP57* (FLAG-ZFP57^Δznf^) which was transiently over-expressed with KAP1-HA. We observed a direct interaction between KAP1 and the ZFP57 mutant lacking all zinc fingers, consistent with the presence of the intact KRAB domain (Figure 3B). This suggests that the LOM we observe in our family is due to a failure of ZFP57^p^^.E^^123*^to binding to DNA. Furthermore, using site-directed mutagenesis we generated a previous published single-base substitution that produces the p.R228H amino acid change (20). This mutation maps to the third zinc-finger and has been shown to reduce the ability to bind methylated DNA (18). This variant also interacts with KAP1 indicating that the MLID in this patient is not due to a failure in recruiting the KAP1 co-repressor complex and is consistent with the observation that the KRAB domain is the primary mediator of KAP1 interactions (Figure 3B). This data points towards a reduced recognition for methylated consensus sequences as the potential mechanism behind MLID.

### ZFP57-KRAB recruitment is sufficient to protect non-imprinted germline-derived allelic methylation from epigenetic reprogramming

**Figure 4.**
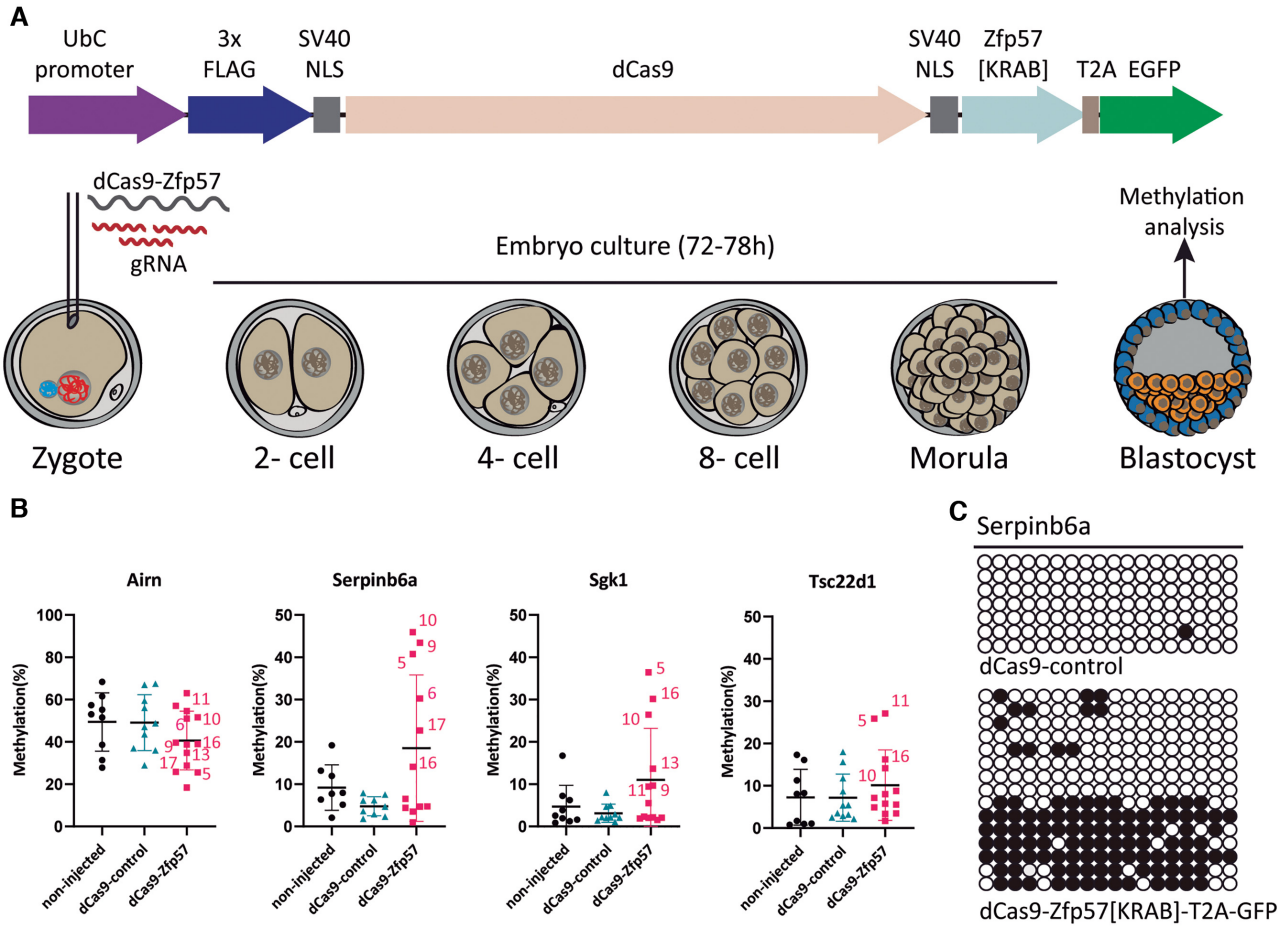
Characterizing the ability of ZFP57 to protect non-imprinted oocyte-derived methylation from epigenetic reprogramming. (**A**) Schematic representation of the dCas9-Zfp57[KRAB]-T2A-GFP construct and the microinjection strategy for mouse zygotes (**B**) The results of nested-multiplex bisulphite PCR coupled with locus-specific pyrosequencing for the maternally methylated imprinted control *Airn* and the targeted loci *Serpinb6a, Sgk1* and *Tsc22d1*. (**C**) Confirmation of strand-specific methylation by bisulphite PCR and sub-cloning for the Serpinb6a interval. Each circle represents a single CpG dinucleotide on a DNA strand, a methylated cytosine (•) or an unmethylated cytosine (○).

We next sought to determine if ZFP57 binding alone is sufficient to recruit the KAP1 co-repressor complex and protect non-imprinted oocyte-derived methylation during pre-implantation development. We hypothesised that the tethering of ZFP57 to genomic locations that are normally methylated in oocytes but reprogrammed very early in pre-implantation development would be sufficient to recruit KAP1 co-repressor complex and maintain allelic DNA methylation. To address this, we generated a dCas9-Zfp57 fusion construct that lacked the zinc-fingers, but retained the full KRAB domain in the -C terminus, ensuring that any DNA targeting was solely due to ribonucleoprotein complex (Figure 4). We validated that our dCas9-Zfp57[KRAB]-T2A-GFP construct could indeed directly interact with mouse KAP1-HA in transfected mouse NIH3T3 cells based on co-IP (Supplementary Figure S3).

To identify specific loci to target, we first interrogated mouse oocytes and embryo RRBS datasets (31) for regions that were highly methylated in oocytes, lack methylation in sperm and progressively demethylate during pre-implantation development. We discarded any genes that presented with a lethal phenotype when knocked-out (according to the Mouse Genome Informatics database) as our manipulations may alter gene dosage in the early embryos. Finally, we selected three different loci, *Serpinb6a, Sgk1* and *Tsc22d1*, to which we designed two crRNAs on opposing DNA strands. The specificity of crRNA targeting was assessed using T7 endonuclease Surveyor assays following transfection of Cas9 and tracrRNA into NIH3T3 cells. All crRNAs were able to target the corresponding regions and, reassuringly, multiplexing did not affect the target efficiency at individual loci (Supplementary Figure S3). Subsequently, *in vitro* transcribed dCas9-Zfp57[KRAB]-T2A-GFP mRNA, multiplexed crRNAs and tracrRNA were microinjected into mouse zygotes. To ensure that any effect we observed was due to the recruitment of the KRAB domain of the dCas9-ZFP57, we also generated a control construct lacking the KRAB effector (dCas9-control) which we also compared to a non-injected control group (Figure 4A). To control microinjections, we took advantage of the ATTO^TM^550 fluorochrome present on the tracrRNA. Embryos which did not display red fluorescence were discarded from subsequent analyses, while positive embryos were developed *in vitro* for 78 h until the blastocyst stage. Individual blastocysts were subsequently used for methylation analysis. We quantified the methylation status of the three targeted regions by utilizing a nested-multiplex bisulphite PCR approach coupled with locus-specific pyrosequencing. To control for amplification bias that may have occurred because of the high number of PCR cycles, we included primers for the *Airn* imprinted gDMR into the nested-multiplex reaction. Following pyrosequencing we observed that two loci, *Sgk1* and *Serpinb6a*, retained methylation in a strand-specific manner upon ZFP57 targeting, suggesting maintenance of oocyte-derived methylation in half of the injected embryos (Figure 4B, C). Two embryos from the *Tsc22d1* experimental group also had elevated methylation levels compared to controls (Figure 4B). Interestingly, the injected embryos that had the highest levels of methylation were the same, suggesting that the amount of ribonucleoprotein complex was sufficient to ensure successful targeting to their intended loci and recruitment of the KAP1 co-repressor complex to non-imprinted loci not normally targeted by ZFP57.

### Alternative *ZFP57* promoter usage originates from a hap-ASM interval

**Figure 5.**
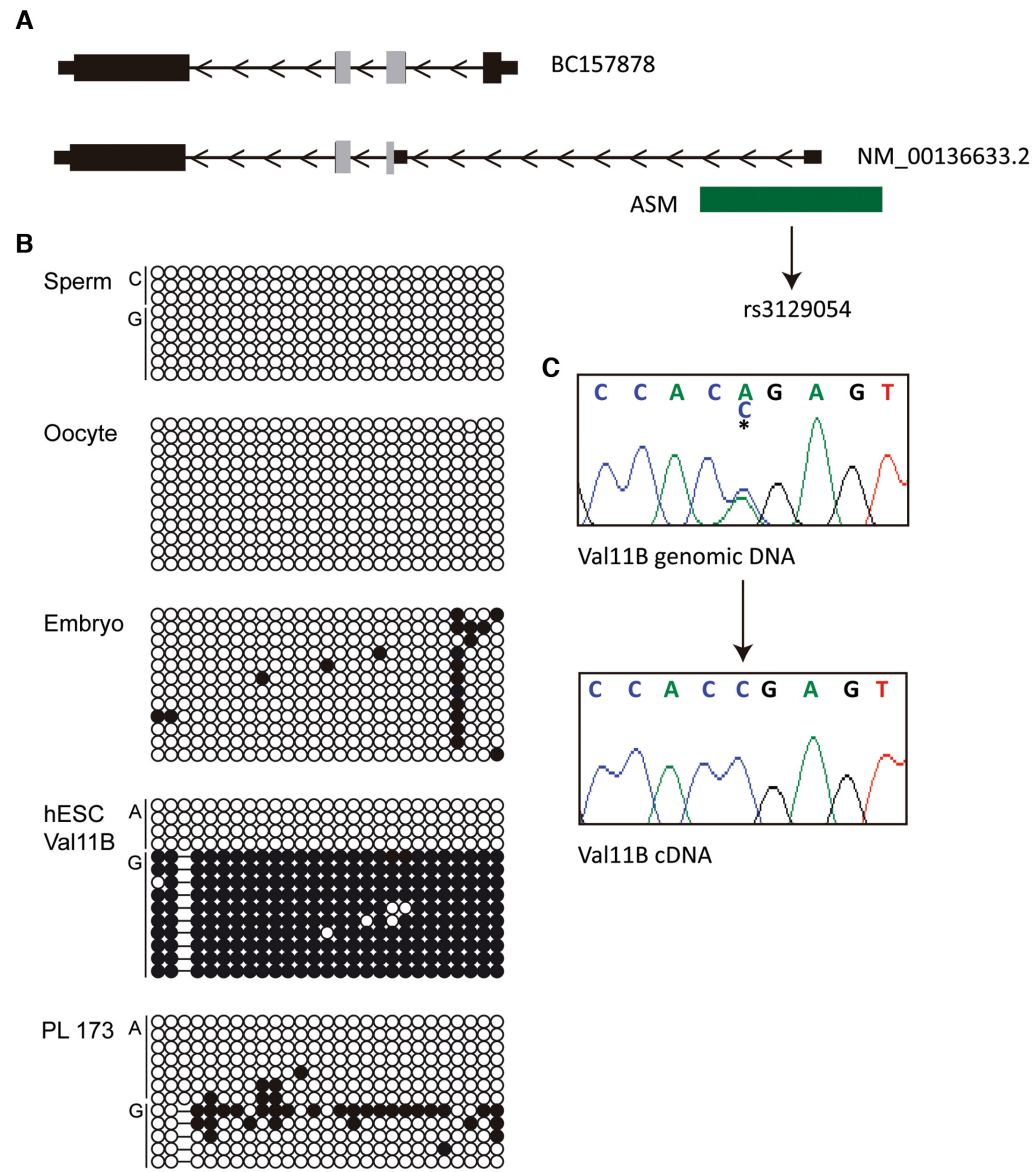
Alternative promoter usage and the *ZFP57* hap-ASM. (**A**) Schematic overview of the *ZFP57* gene, the location of two transcripts observed in blastocyst -derived RNA-seq datasets and the position of the hap-ASM. Grey exonic regions indicate the KRAB domain sequences. (**B**) Characterization of the allelic expression in Val11B hES cell line and methylation overlapping the alternative *ZFP57* promoter heterozygous for SNP rs3129054 by bisulphite PCR followed by sub-cloning. Each circle represents a single CpG dinucleotide on a DNA strand, a methylated cytosine (•) or an unmethylated cytosine (○). (**C**) Characterisation of monoallelic expression in the Val11B hES cell line.

Mutations of mouse *Zfp57* cause the resulting phenotypes via maternal-zygotic effect and are directly associated with high oocyte *Zfp57* expression, whereas the phenotypes in humans are only apparent following recessive transmission and the inheritance of two mutated alleles. To determine if this species-specific difference in inheritance pattern is linked to the developmental timing of expression, we determined *ZFP57* expression in our RNA-seq datasets from human oocytes, cleavage embryos and blastocysts. This revealed that human *ZFP57* is not expressed in oocytes and is only detectable at the blastocyst stage (Supplementary Table S4). Interrogation of the aligned sequences revealed alternative promoter usage due to the identification of a unique first exon (NM_001366333.2). This ∼90 bp 5’UTR sequence is located 4 kb upstream of the first exon of the most prevalent isoform (BC157878) in an interval previously reported to show haplotype-dependent allele-specific methylation (hap-ASM) in T-cells (32) (Figure 5A). We confirm methylation asymmetry between the two alleles in a heterozygous hES cell line (Val11B), which resulted in monoallelic expression of *ZFP57*, but methylation is absent in gametes and term placenta (Figure 5B, C). Quantitative RT-PCR with exon-spanning primers revealed a similar expression profile for both isoforms, indicative of co-expression in most tissues, being most abundant in spine, heart, hES cells and placenta (Supplementary Figure S4). Fascinatingly, the isoform originating from the alternative promoter results in skipping of exon 2 and a significantly truncated KRAB domain (Figure 5A).

### Temporal expression profiling identifies *ZNF202* and *ZNF445* potential imprint factors

**Figure 6.**
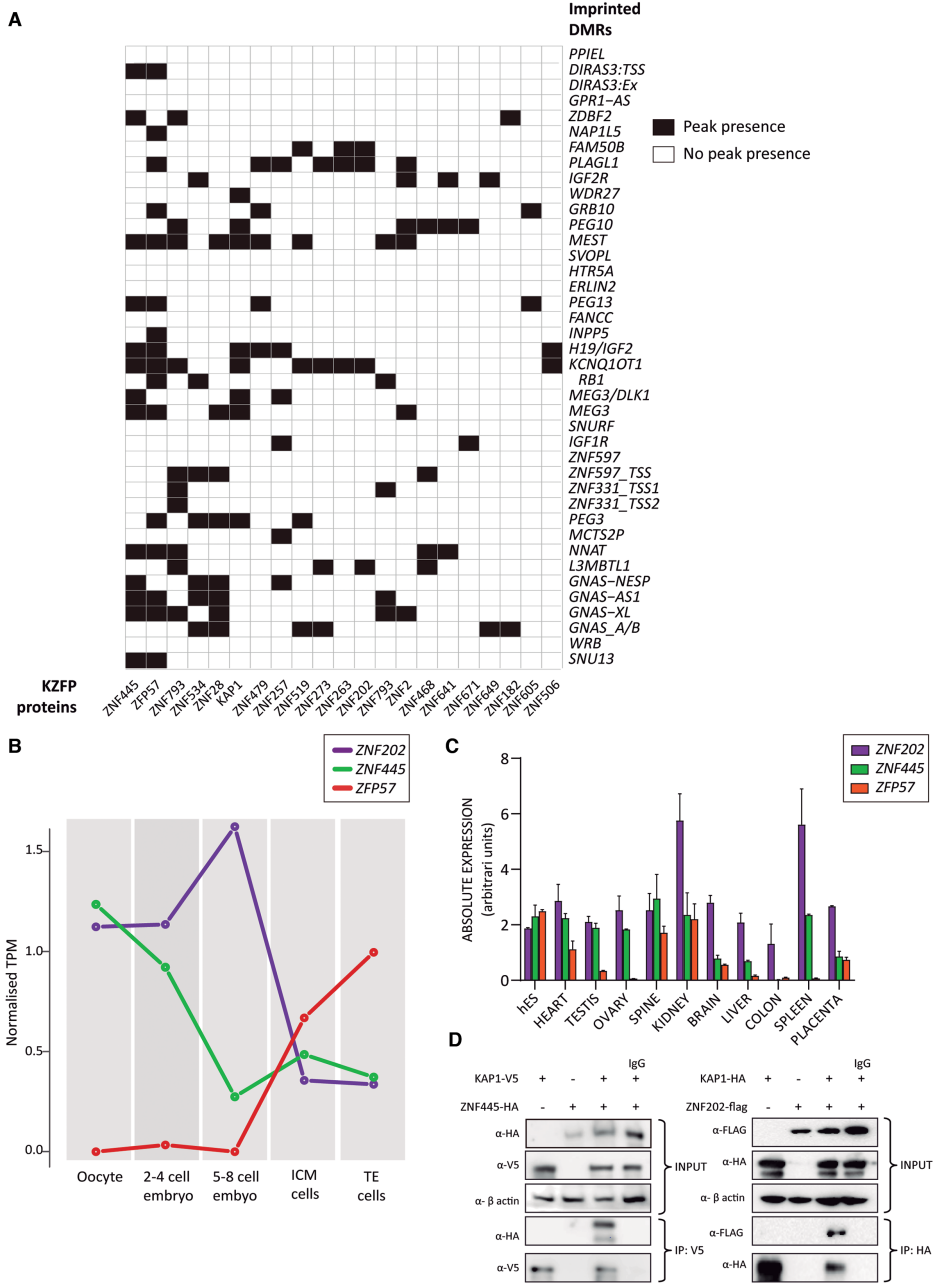
Identification of candidate KZFPs binding to imprinted DMRs. (**A**) Graphical representation of interactions between KZFP and multiple imprinted DMRs. White squares indicate a lack of binding, whereas black squares represent a positive ChIP-seq peak located within the defined imprinted DMR sequence. (**B**) RNA-seq expression profile for *ZNF202, ZNF445* and *ZFP57* during human pre-implantation development. (**C**) Quantitative RT-PCR showing the distribution of expression for the same three KZFPs across different tissues. (**D**) Western blotting of co-immunoprecipitations between FLAG-ZNF202 and HA-ZNF445 with epitope tagged KAP1.

Our RNA-seq profiling revealed that human *ZFP57* is only detectable following embryonic-genome activation (EGA) suggesting that other factors may recruit the KAP1 co-repressor complex to maintain methylation at imprinted gDMRs during the cleavage stages of development which coincided with epigenetic reprogramming. To determine if other KZFPs could be involved in this process we questioned whether any of those with available high-resolution KZFP ChIP-seq maps, could bind to ubiquitously imprinted DMR. Recently ChIP-seq for 223 KZPs (12), representing ∼30% of all KZFPs in the human genome has been reported. The analysis exposed 49 KZFPs binding to different imprinted DMRs, with 25 binding to multiple loci (Figure 6A; Supplementary Figure S5 and S6). Focusing on the 25 potential candidates that can bind to multiple imprinted loci, we next filtered candidates based on their expressed profile, which we limited to before EGA and the expression of *ZFP57*. For this purpose, we re-interrogated our RNA-seq datasets. Only two KZFPs were highly abundant in oocytes and expressed during the first cleavage divisions; *ZNF202* and *ZNF445* (Figure 6B). Quantitative RT-PCR for these two factors revealed ubiquitously expression in most tissues (Figure 6C). Both genes possess SCAN motifs in the N-terminal domain upstream of their KRAB domains, with ZNF202 having eight zinc fingers and ZNF445 fourteen. Using transient overexpression, ZNF202-FLAG and ZNF445-HA both interact with KAP1 in co-IP experiments confirming their ability to recruit the KAP1 co-repressor complex (Figure 6D).

Additional analysis of the high-resolution KZFP ChIP-seq maps revealed that ZNF202 binds to four imprinted DMRs (*FAM50B, PLAGL1, KCNQ1OT1 and L3MBTL1*), as well as >7000 addition loci throughout the human genome (12). This implies that ZNF202 has no particular selectivity for imprinted domains. On the contrary, ZNF445 binds to 13 different imprinted DMRs (*DIRAS3, ZDBF2, MEST, PEG13, H19, KCNQ1OT1, MEG3-DLK1* IG-DMR, *MEG3, NNAT, GNAS-NESP, GNAS-AS1, GNAS-XL and SNU13*) out of a total of 638 genome-wide locations. When comparing these imprinted loci to those bound by ZFP57, we found that 77% (10/13) are in common, suggesting these two proteins could work in unison to protect the imprinted regions during early development.

### Screening MLID cohorts for *ZNF202* and *ZNF445* mutations

Since very few mutations in epigenetic factors have been directly associated with MLID, we performed a direct screen for pathogenic variants that could act via maternal-effect in ID patients with Beckwith-Wiedemann syndrome (BWS), Pseudohypoparathyroidism type 1B (PHP1B) and TNDM, along with their mothers. Amplification of *ZNF202* and *ZNF445* exons and direct Sanger sequencing of the resulting amplicons identified one heterozygous *ZNF445* variant (rs143141433) in the mother of a child with PHP1B MLID that was not present in 200 controls. This rare missense variant (1000 genomes 36/5008) results in a p.E957G amino acid change that was predicted to be benign following Polyphen analysis.

## DISCUSSION

Multi-locus imprinting disturbances have been observed for most IDs with varying frequency, often mirroring the incidence of epimutation for each individual disease. Despite intense efforts, the underlying genetic causes have rarely been identified, with mutations in *ZFP57* and members of the SubCortical Maternal Complex (SCMC) being recurrently observed in different patient cohorts (20,33–35). Intriguingly, mutations in *ZFP57* are associated with TNDM and a highly predictable methylation signature (20–23), whereas there is considerable variability in both clinical presentation and imprinted loci affected in cases with underlying SCMC involvement.

In contrast to mouse where ZFP57 maintains allelic methylation at the majority of gDMRs (14–16), humans are less dependent on this KZFP. Using extremely rare samples from a family harbouring a recessive *ZFP57* mutation we investigated the influence of this KZFP on imprinted gDMR methylation. We show that the truncating mutation leads to absolute hypomethylation of *PLAGL1* DMR and varying degrees of LOM at several additional imprinted loci which is reproducible, not only between tissues of an affected individuals, but stable over time. Furthermore, the affected sibling also had similar aberrant methylation profiles. The imprinted gDMRs associated with *NAP1L5, GRB10* and *GNAS* presented incomplete LOM in these subjects, which we were able to show was due to cell mosaicism rather than allelic partial demethylation.

Studies in mouse models, both *in vivo* and in mES cells, have shown that ZFP57 binds to the methylated allele at gDMRs and recruits the scaffold protein KAP1, the obligatory histone methyltransferase SETDB1 and DNA methyltransferases to deposit H3K9me3 and cytosine methylation (5mC) at target sequences (13,15). However, it is unknown whether human ZFP57 recruits these and other components of the co-repressor complex. Using co-IPs, we show that human ZFP57 does indeed interact with KAP1, DNMT3A/B and HP1γ. These observations endorse the model centred around a sequence-specific KZFP recruiting KAP1, which in turn encourages a self-perpetuating loop in which SETDB1 deposits H3K9me3 that is recognised by HP1. Furthermore, since ZFP57 has a strong preference for methylated DNA the inclusion of DNMT3s within the co-repressor complex safeguards the underlying 5mC. In general, methylation is heritably maintained in somatic cells by the Uhrf1/Dnmt1 complex that recognizes hemi-methylated DNA and methylates the newly synthesized unmethylated daughter strand, protecting against methylation dilution during each round of DNA replication (36). This form of maintenance is different to that occurring during pre-implantation reprogramming, which is largely dependent upon the sequence-specific recruitment of the co-repressor complex, which is consistent with our observations that ZFP57 interacts with the DNMT3A/B and not DNMT1. Interestingly, both the ZFP57^p.E^^123*^deletion and the non-synonymous p.R228H base substitution retain the ability to interact with KAP1, suggesting that if present in the cell of patients, these mutated forms would bind KAP1 but would not recruit the co-repressor complex to imprinted gDMRs. Scrutiny of *ZFP57* expression in blastocysts identified an alternative splice isoform that would result in the translation of a protein lacking the KRAB domain, and therefore would not have the capacity to recruit KAP1, despite binding to target sequences. Similar isoform structure has been noted for several additional KZFPs, including *ZNF74, ZNF468* and *ZNF268* (37–39), with competitive repressive action occurring between isoforms, with the KRAB-containing version responsible for silencing transcription. If this antagonism occurs for ZFP57 in the TE and placenta, it may result in widespread LOM accounting for the polymorphic nature placenta-specific imprinted gDMRs in humans. More work is required to follow-up on this hypothesis.

Curiously ChIP-seq data generated in HEK 293 cells by Imbeault and colleagues reveals that human ZFP57 binds to several more imprinted gDMRs than are affected in the TNDM MLID probands (12). One possible reason for such a discrepancy could be that starker LOM only occurs following maternal-zygotic transmission, such as in mouse, since most cases in humans are zygotic mutants. However, this is unlikely, since a TNDM MLID case with a homozygous *ZFP57* mutation was born to a homozygous mother, who herself presented with TNDM at birth. The child had no evidence of a more severe phenotype nor was the degree of her LOM more severe than other individuals with *ZFP57* mutations (21). An alternative explanation is that there is compensation by other KZFPs that cooperate to maintain imprinted gDMRs and that the affected loci in *ZFP57*-mutated patients would not be bound by the additional KZFPs. Such cooperation between KZFPs is also supported by the fact that *ZFP57* is not expressed during the critical time just after fertilization in humans, implying other KZFPs are necessary to initially recruit the KAP1 co-repressor complex to imprinted gDMRs. Since our oocyte and embryo RNA-seq datasets confirmed this notion, we bioinformatically analysed the publicly available high-resolution KZFP ChIP-seq maps to identify addition candidates that bind to imprinted DMRs. Whilst many KZFPs bind to these genomic intervals, very few are associated with multiple imprint loci. Furthermore, when we determined the correct temporal expression in our human RNA-seq datasets, only two KZFPs were highly abundant in oocytes with continued expression during cleavage stages of development, *ZNF202* and *ZNF445* respectively. Since ZNF202 only bound to four imprinted DMRs and promiscuously binds throughout the genome (12), we focused our attentions on *ZNF445*.

Recently Takahashi and co-workers identified *ZNF445* as a primary regulator of genomic imprinting in mouse (40). Although maternal-zygotic deletions of *Zfp445* in mouse embryos did not affected imprinted DMRs or allelic expression, double homozygous zygotic mutations for *Zfp445* and *Zfp57* gave rise to more remarkable phenotypes associated with dramatic LOM than either *Zfp445* or *Zfp57* mutations alone. This led the authors to conclude that murine imprinted DMRs depend on ZFP445 and ZFP57 to different degrees and that these KZFPs act in unison to maintain allele-specific imprints in early mouse development. Our human expression data suggests that rather than acting together during the epigenetic reprogramming window, these two KZFPs separately recruit the KAP1 co-repressor complex, ZNF445 initially, followed by ZFP57, since they are not co-expressed. We speculate that pathogenic mutations in *ZNF445* would not be compatible with life, since a large number of imprinted gDMRs could present with LOM during early development. However, since individuals with biallelic *ZFP57* mutations survive (maybe with an ascertainment bias for those with the DMR signature reported), as do mice carrying targeted *Znf445* deletions, mutations in *ZNF445* may have no clinical consequence. To decipher whether mutations of *ZNF445* are associated with developmental phenotypes it may be more appropriate to screen women with idiopathic infertility or recurrent miscarriage in the future.

In conclusion, our studies have identified developmental and epigenetic discrepancies between the role of ZFP57 in mouse and humans which are due to difference in the timing of expression during pre-implantation development. Screening for additional KZFPs expressed during this crucial developmental window identified ZNF445, which works concurrently with ZFP57 to recruit the KAP1 co-repressor complex in mouse, but unaccompanied immediately following fertilization in humans. Our analysis also identified other KZFPs with the capacity to bind individual imprinted gDMRs, suggesting that they may be candidates for isolated LOM or milder cases of MLID observed in IDs.

## DATA AVAILABILTY

The coordinates of human imprinted gDMRs used in this are available at www.humanimprints.net. The IIIumina Infinium HumanMethylation450 and HumanMethylationEPIC BeadChip array data has been deposited in the GEO repository and assigned the accession number GSE149568. The RNA-seq expression data corresponding to the oocyte and embryo datasets can be found at NCBI-BioProject under the accession number PRJNA630371.

## Supplementary Material

gkaa837_Supplemental_Files
