# Supplementary material for: The role of ZFP57 and additional KRAB-zinc finger proteins in the maintenance of human imprinted methylation and multi-locus imprinting disturbances
Source: Nucleic Acids Res. 2020 Oct 14;48(20):11394–407. doi: 10.1093/nar/gkaa837 (PMC7672439; doi:10.1093/nar/gkaa837)
Supplement: gkaa837_Supplemental_Files [file gkaa837_supplemental_files.zip › Supplementary material_Figures_Ammended.pdf]

SUPPLEMENTARY MATERIAL

Table S1. List of PCR primers used in this study.

Table S2. List phenotypic features for TNDM-MLID patients II.1 and II.2.

Table S3. The absolute methylation values (β-values) for individual probes mapping to 35 ubiquitously imprinted DMRs in patient samples and control leukocytes (EPIC II.1 and II.2 samples, HM450k for control and I.1).

Table S4. Oocyte and embryo scRNA-seq expression profiles (TPM) for *ZFP57, ZNF445* and *ZNF202*.

Figure S1

(A)

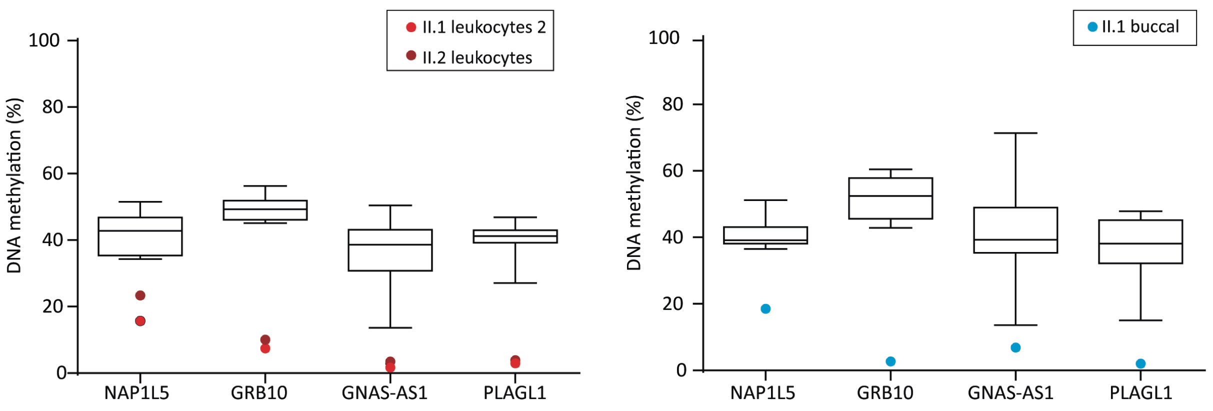

(B)

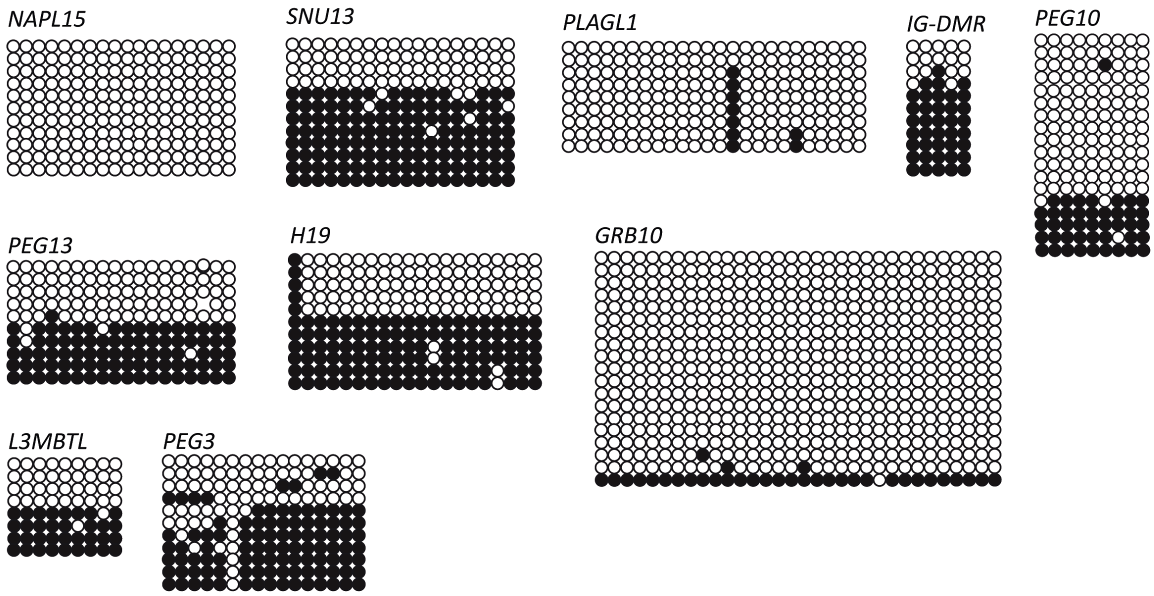

Methylation profiles of *ZFP57*$^{p.E123*}$ probands as determined by pyrosequencing and bisulphite PCR and sub-cloning. (A) Quantitative pyrosequencing of four imprinted DMRs in leukocytes and buccal-derived DNA. The boxplot showing the median methylation (whiskers 5–95% percentile) determined for 15 control DNA samples and the values of II.1 (original and repeated sampling) and II.2 highlighted as shaded circles. (B) Confirmation of LOM at imprinted DMRs in II.1 leukocyte 1 by bisulphite PCR and sub-cloning. Each circle represents a single CpG dinucleotide on a DNA strand, a methylated cytosine (⬤) or an unmethylated cytosine (○).

Figure S2

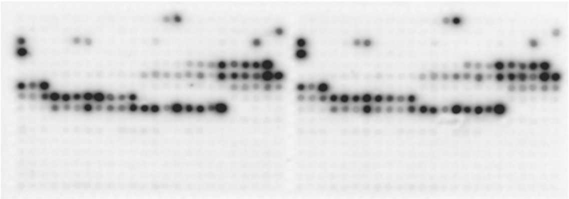

HP1γ histone peptide array

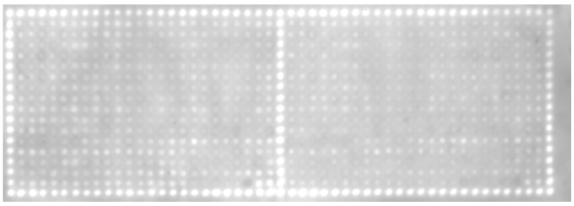

ZFP57 histone peptide array

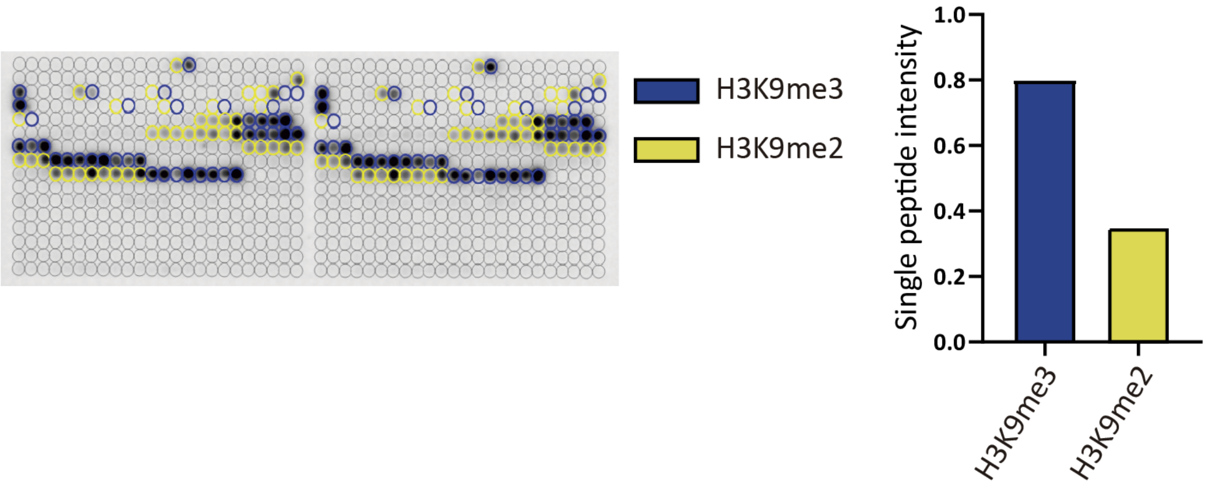

MODified histone peptide array analysis for ZFP57 and HP1γ binding. Arrays were incubated with the corresponding purified proteins and detection antibodies. Black dots depict binding between a histone modification and the protein of interest. Quantification of HP1γ reveals interaction with H3K9me2 and H3K9me3.

Figure S3

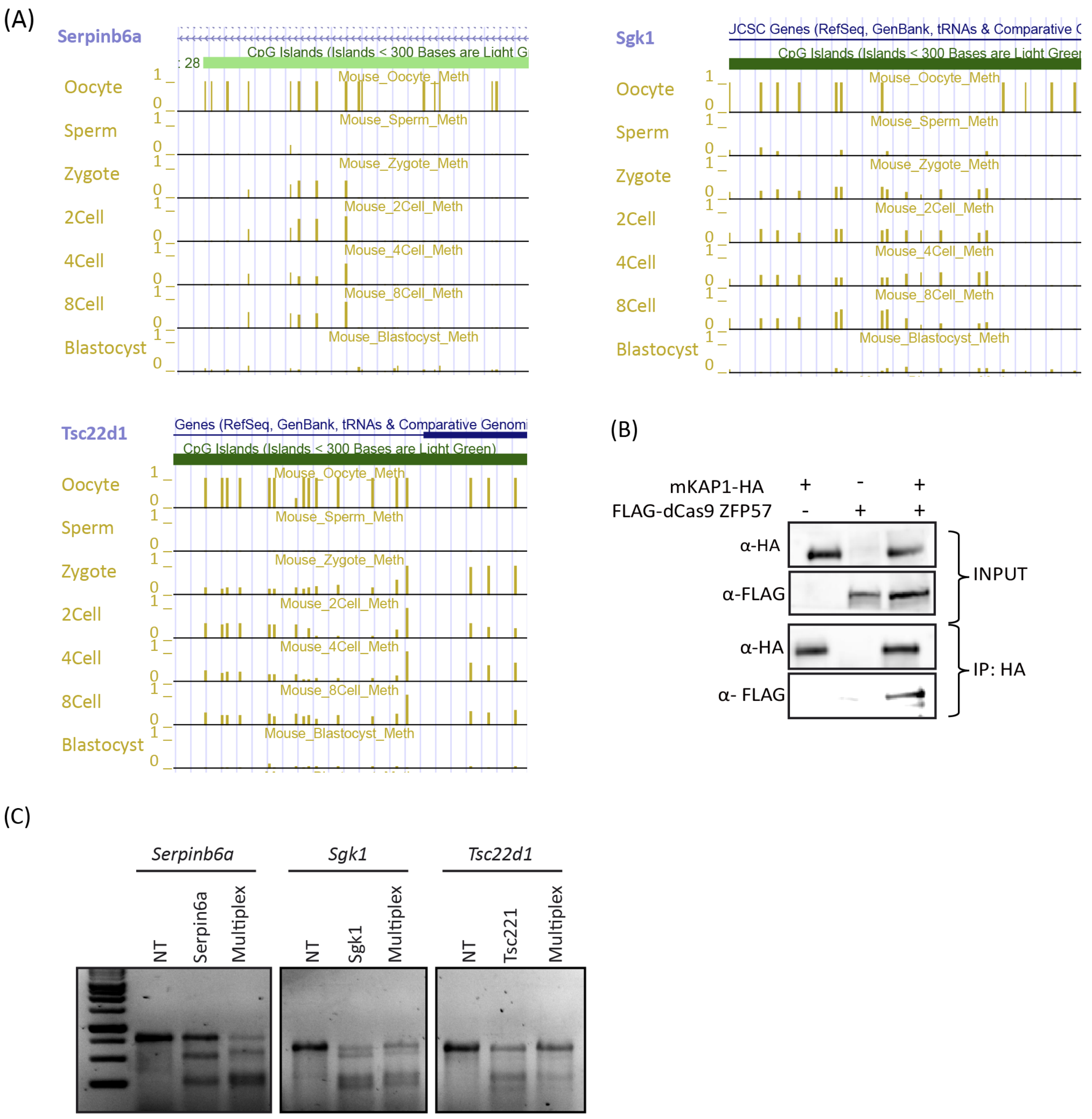

Selection of loci with non-imprinted oocyte-derived methylation and the optimization of the dCas9-Zfp57[KRAB]-T2A-GFP construct and crRNAs. (A) The methylation profiles for *Serpinb6a, Sgk1* and *Tsc22d1* in developmental RRBS datasets. (B) Co-IP between the dCas9-Zfp57[KRAB]FLAG-T2A-GFP and mouse KAP-HA in NIH3T3 cells. (C) T7 endonuclease assays performed in NIH3T3 cells transfected with wild-type Cas9, tracrRNA and combinations of crRNAs. The presence of multiple bands indicates that the ribonucleoprotein complex is targeted to each locus.

Figure S4

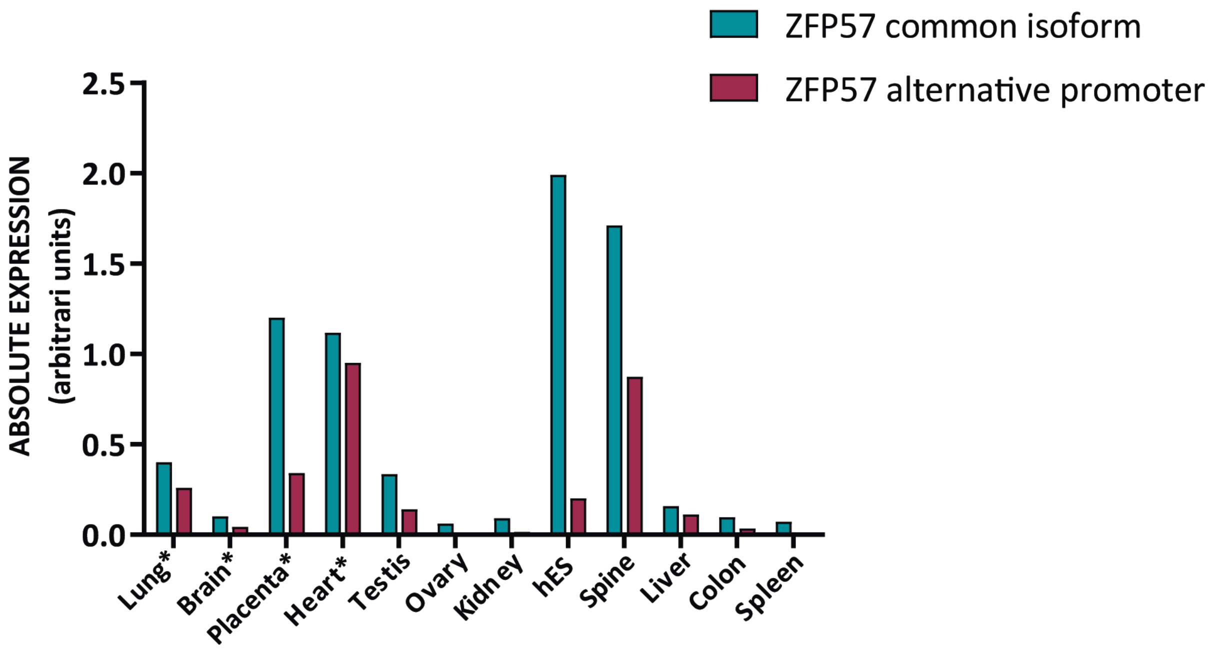

Expression profiling of the two *ZFP57* isoforms in tissues. Quantitative RT-PCR showing the distribution of expression across different fetal and adult tissues. Tissues marked with (*) are of fetal origin.

Figure S5

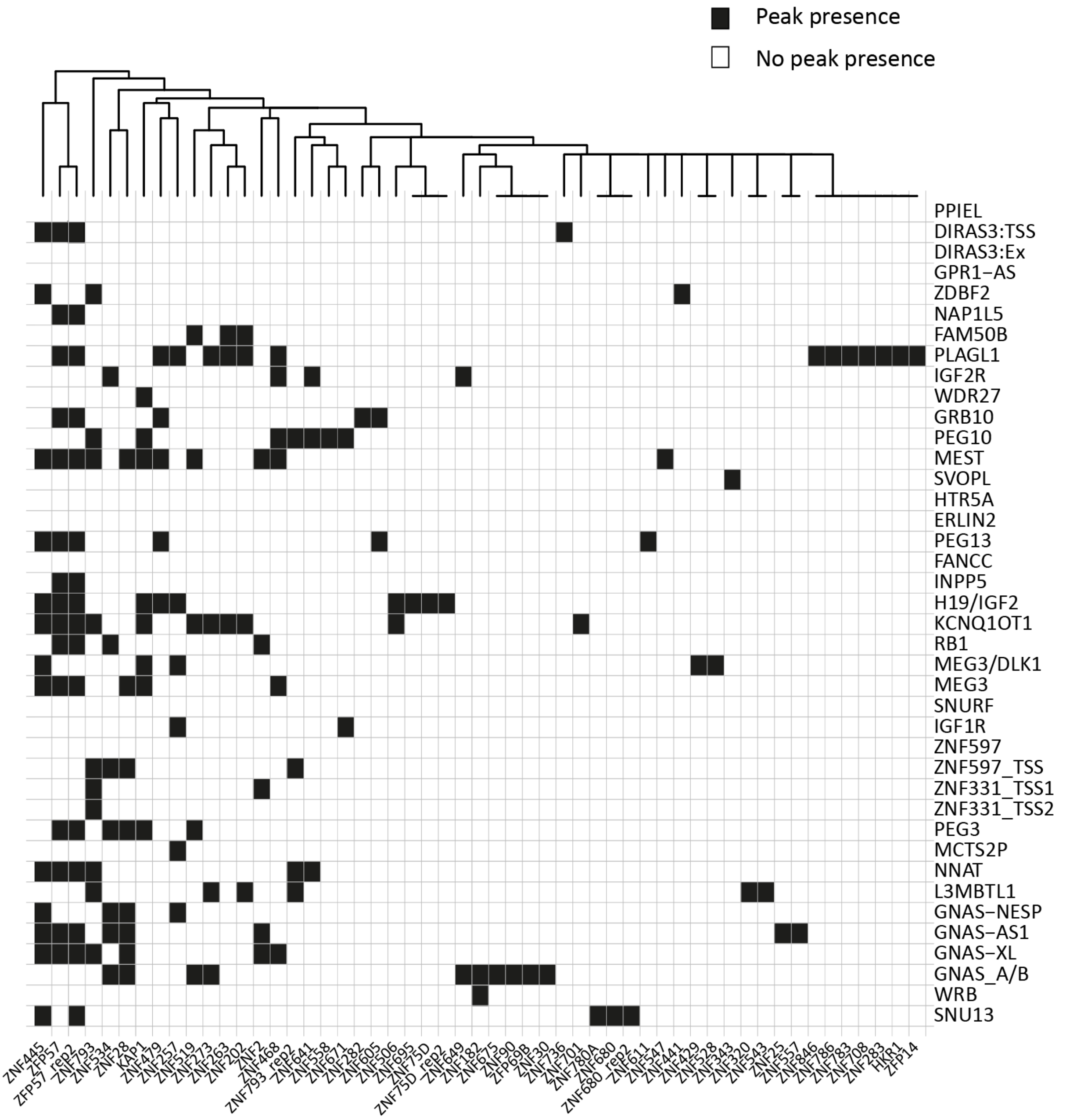

Schematic map of KZFP binding to all imprinted DMRs. Graphical representation showing the full extent of KZFP binding to single and multiple imprinted DMRs. White squares indicate a lack of binding, whereas purple squares represent a positive ChIP-seq peak located within the defined DMR sequence.

Figure S6

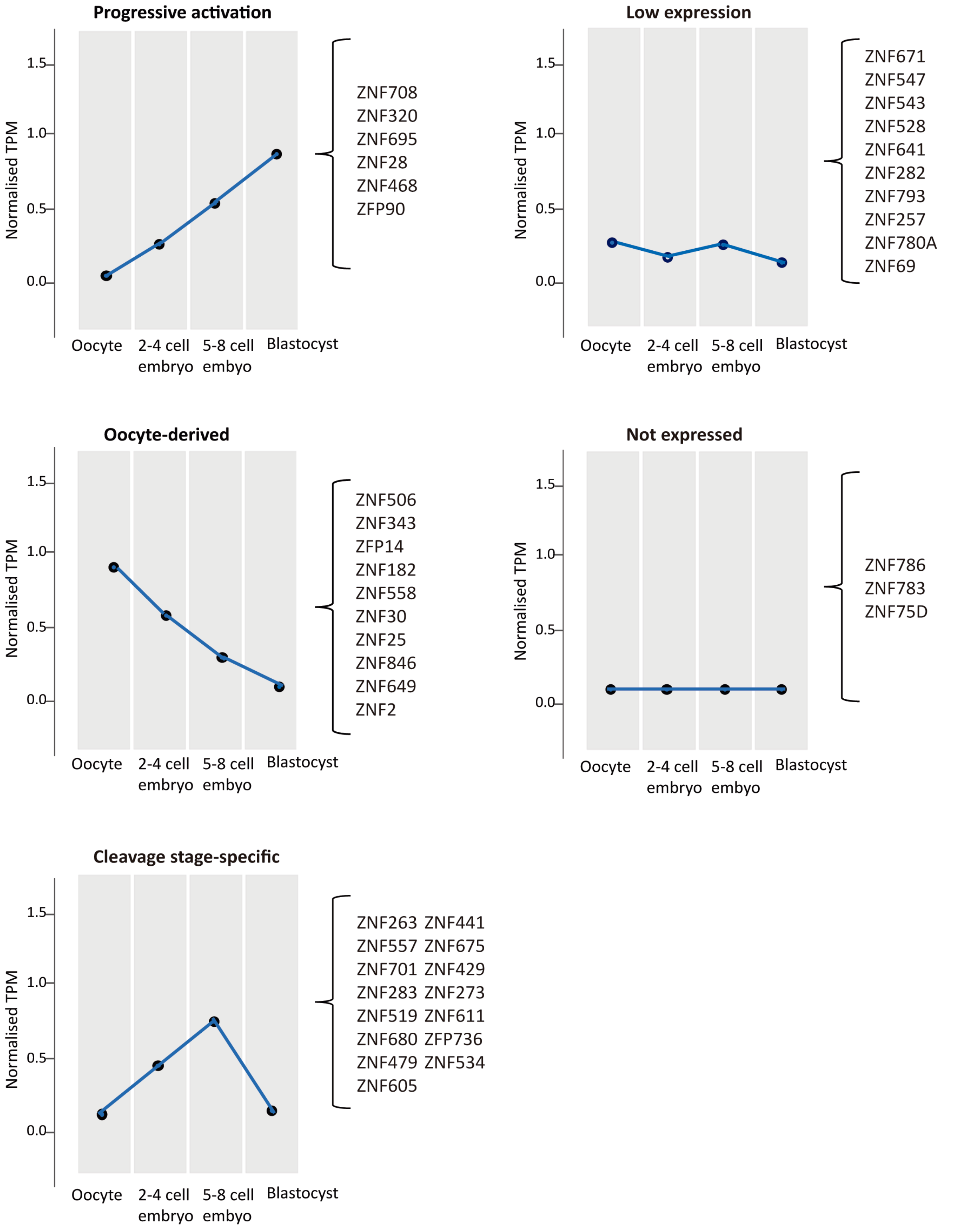

Early developmental expression profiles for KZFPs binding to one or more imprinted DMRs. Grouped as those with progressive transcriptional activation, oocyte-derived decline, cleavage stage specific activation, low variable or not expressed.
